# Tracking Secondary School Students: A Qualitative Investigation of an Online Student Monitoring System With Parent Portal

**DOI:** 10.1111/josh.70097

**Published:** 2025-12-02

**Authors:** Eline M. Meuleman, Ina M. Koning, Danique M. Heemskerk, Vincent Busch, Maartje M. van Stralen

**Affiliations:** ^1^ Department of Health Sciences, Faculty of Science and Amsterdam Public Health Research Institute Vrije Universiteit Amsterdam Amsterdam the Netherlands; ^2^ Department of Educational and Family Studies, Clinical Child and Family Studies Vrije Universiteit Amsterdam Amsterdam the Netherlands; ^3^ Department of Healthy Living The Public Health Service of Amsterdam (GGD Amsterdam) Amsterdam the Netherlands

**Keywords:** educational technology, parent–child relationship, secondary school, student monitoring systems, student wellbeing

## Abstract

**Background:**

The transition to secondary school brings academic and social challenges, with student monitoring systems offering real‐time insights to enhance communication between students, parents, and teachers. While these systems improve tracking and support, their effects on student autonomy, wellbeing, and parent–child dynamics are understudied.

**Methods:**

This exploratory research examines the perspectives of five students, seven parents, and twelve teachers using Dutch secondary school systems with parent portals, by conducting semi‐structured interviews.

**Results:**

Results of the interviews show benefits such as improved teacher communication and better parental support for students but also challenges such as increased teacher workload and concerns over student privacy, autonomy, and well‐being.

**Implications for School Health Policy, Practice, and Equity:**

Schools should balance the benefits of student monitoring systems with their impact on autonomy and well‐being. While progress tracking is valuable, unrestricted parental access may lead to over‐monitoring. Limiting access to designated times (e.g., four times a year) can preserve student independence while keeping parents informed.

**Conclusion:**

The study highlights the need for balanced system design to protect students' wellbeing and autonomy while enhancing educational collaboration.

## 1 Introduction

The transition from primary to secondary school brings significant changes for students and parents. Students must adapt to a new social and academic environment with increased homework and assessments, which are often tracked via student monitoring systems (SMS) [1, 2, 3]. Originally used by teachers to record grades and track progress, these systems now offer students and parents 24/7 access to grades, assignments, and attendance through digital apps, facilitating parental involvement [4]. Although parental involvement typically decreases as children transition from primary to secondary school [5, 6], these systems keep parents constantly informed. However, as the exact benefits and drawbacks of these systems are still unclear, research is needed to understand how this constant access to information about school‐related issues affects students, parents, and teachers.

This availability of online SMS with parent portals wherein information on students' grades, attendance, evaluations, and classroom activities is continuously shared can come with benefits and drawbacks for the stakeholders involved. Some of the advantages are: (1) they save teachers time by automatically updating student data, (2) they assist in monitoring student progress, and (3) they promote greater parental involvement [4]. Timely access to information about student grades and missed assignments enables teachers and parents to provide the necessary support, which can be crucial for reducing course failures and increasing class attendance [7].

However, for students, an online monitoring system with a parent portal may also have disadvantages. Firstly, it may negatively impact their wellbeing since students are 24/7 digitally connected to their school; homework, class schedules, and grades [8]. Secondly, parent portals may cause a reduced sense of autonomy, as students may feel a lack of privacy [4]. Based on the Self‐Determination theory [9], the need for autonomy increases in the transition from primary to secondary school, where autonomy‐granting parenting is beneficial for child development [10, 11]. Autonomy‐supportive parenting behaviors are attuned to the child's needs, preferences, and interests, while encouraging their own initiatives and providing room for independent decision‐making [12]. Providing parents with continuous insight into school activities may foster a parenting style that undermines autonomy, known as “overparenting,” that is parenting characterized by overprotection, overcontrol, and an excess of involvement [13]. This way of parenting potentially limits opportunities for children to experience and cope with everyday challenges and could result in a failure to develop essential skills, such as problem‐solving abilities and/or functional coping strategies [14]. Overparenting has been linked to lower levels of a teenager's self‐control which impairs their subsequent wellbeing and has been associated with higher chances of experiencing school burn‐out [15]. It is thus likely that the parent portal as part of SMS, may interfere with adolescents' development of autonomy and may contribute to a lower level of wellbeing.

The evidence described above for the various advantages and disadvantages, comes from research with a focus on other content areas (such as sleep, automated communication technologies or online learning) or from online discourse analyses. To the best of our knowledge, the perceptions of different stakeholders on the advantages and disadvantages of the use of always‐available, real‐time SMS during secondary school and its impact on students' wellbeing and the parent–child relationship remain understudied. Hence, the aim of this study was to adopt a qualitative exploratory design to examine how different stakeholders—students, parents, and teachers—experienced the use of an SMS with a parent portal in a specific context, namely the first and second years of Dutch secondary education.

## 2 Methods

### 2.1 Participants

This study employed a convenience sampling strategy, with recruitment conducted by university students completing (teaching) internships at various secondary schools across the Netherlands. Participants (students, teachers, and parents) were recruited from the personal networks available to these university students within the schools where they were undertaking their internships. The following inclusion criteria were applied for each participant group.

Students had to be in their first or second year of Dutch secondary school (aged 12–14), representing different educational tracks (*vmbo*, *havo*, *vwo*). All students were required to speak Dutch. Students receiving special education or attending alternative educational settings were excluded. Caretakers had to be the parent (mother or father) of a student in the eligible age and school range. Only Dutch‐speaking caregivers were included. Teachers had to be currently teaching first‐ and/or second‐year students at a Dutch secondary school where recruitment took place. No restrictions were placed on teaching subject or years of experience.

### 2.2 Instrumentation

Semi‐structured interviews of approximately 30 min were conducted either face‐to‐face or online, depending on the participant's preference. The semi‐structured interviews were based on a topic list that consisted of questions on the use of SMS, its advantages and disadvantages, and the influence of such a system on students' development, among other factors (see Table 1).

**TABLE 1:** Overview of topics in the semi‐structured interview.

Topic	Questions
Use of the SMS (SMS)	How does your school use the SMS? Do you know if this system is different from the one used by teachers in primary schools?
Advantages and disadvantages	Can you name some general advantages of the SMS? What advantages does the SMS have for you? Can you name some general disadvantages of the SMS? What disadvantages does the SMS have for you?
Perceived impact on the relationship between parents and children	Can you imagine that the SMS influences the relationship between parents and children? To what extent do parents consult the SMS to view their children's grades? Do you think the SMS affects the autonomy of children?
Perceived impact on student wellbeing	To what extent does the SMS, in your perception, affect the wellbeing of students/affect your wellbeing (student question)?
Other consequences	Does the SMS have other consequences?

### 2.3 Procedure

Participants were invited to participate by the research assistants involved in the project and those conducting the interviews. Before the interview, participants were briefed on the study's purpose, assured of their anonymity, and informed that they could withdraw at any time without explanation. Active informed consent was obtained by all participants, and additional active parental consent was obtained for adolescents. After completing the interview, participants received a voucher (15 euros) as reimbursement.

### 2.4 Data Analysis

Interviews were audio‐taped and subsequently transcribed verbatim. The data were analyzed using qualitative analysis methods, by using the thematic analysis technique by Braun and Clarke [16]. Qualitative data analysis software (MAXQDA 2024) was used to support coding, organizing, and selecting the data from transcripts.

As a first step, a calibration session was held in which four researchers independently read and discussed three interview transcripts (one of a student, one of a parent, one of a teacher). Based on this discussion, an initial codebook was collaboratively and inductively developed, capturing preliminary themes and codes emerging from the data. The coding process then proceeded iteratively: one researcher began coding a portion of the transcripts using the initial codebook and refined it based on new insights. Each subsequent researcher used the updated version of the codebook to code their assigned transcripts, further refining it as needed. All transcripts were divided among the four researchers and coded independently using this iterative, collaboratively developed codebook. To ensure consistency and reliability beyond this iterative coding process by multiple researchers, a fifth researcher reviewed all coded segments across the dataset.

## 3 Results

In total, 24 participants were interviewed, including 12 teachers (age 23–56 years), seven parents (41–55 years), and five students (12 and 13 years). The gender of the participants was not reported. See Table 2 for a summary of the key themes and representative quotes.

**TABLE 2:** Summary of key themes and quotes.

Theme	Summary of theme	Representative quote
SMS use	SMS offers real‐time access to grades, schedules, and homework, unlike the periodic reports used in primary school.	“In primary school, it was just brief notes. Now it's more detailed reports teachers can add.”
Teachers' workload	Teachers experience: (1) increased workload due to detailed reporting and expectations of 24/7 availability, and (2) reduced workload through automation and improved communication.	“It's easy to track attendance, homework, and spot concerns across students to monitor their development.”
Teachers' expectations	Teachers form early expectations based on past records. Teachers expect students to be less forgetful and parents to share responsibility in monitoring progress.	“A student can't get away with saying they didn't know. Because you put it in the system”
Students' competence	SMS helps students stay organized and gain support of teachers and parents in the short term but may reduce long‐term development of planning and responsibility skills.	“Students rely less on their own agendas and more on the system.”
Students' wellbeing	While SMS can provide reassurance and support, it can also increase stress due to constant grade notifications and pressure.	“Students get stressed when grades pop up late at night.”
Students' autonomy	Continuous parental access may limit students' ability to manage their own learning and make independent choices.	“Helicopter parenting prevents children from taking responsibility.”
Parent–child relationship	SMS enables parents to support learning more effectively, but can also lead to tension, especially when grades are poor.	“Some students ask teachers to delay grade posting to avoid conflicts.”

### 3.1 Perceived Experience of SMS Use

At all participating schools, a student monitoring system (SMS) was actively in use. Stakeholders noted that this system differed significantly from what they had experienced in primary school, where student progress was typically shared through report cards issued a few times per year. In contrast, the SMS used in secondary school provided real‐time updates on students' grades, attendance, and homework. One teacher said: “Yes, and I think that's different from primary school. There, you often write small notes briefly, whereas here you have more detailed reports that the teacher can add.”

All stakeholders stated that SMS serves multiple purposes. It allows teachers to easily manage their students' grades, track their behavior and attendance, monitor their development, and communicate updates regarding schedule changes, homework assignments, and grades. Students and parents use the system for reviewing schedule changes, homework, and grades, and for communicating with teachers. The use of these features was experienced by all interviewed stakeholders as having both benefits and drawbacks, which are explored in detail below.

### 3.2 Impact on Teachers' Workload

Teachers discussed how SMS impacts their workload. On one hand, keeping all student administration in the system was considered time‐consuming. This has to do with the level of detail of the report, as well as the language that ensures privacy, which should be used. A teacher spoke about how all reports must be suitable to make public in the system upon parents' request, which means teachers spend more time on word choices, sentence structure, and nuances. In addition, teachers are expected by the students and parents to timely insert all grades, homework, feedback, and timetables into the system. This creates the assumption that teachers should continuously monitor and report on every detail of each student's progress. Moreover, teachers indicated that some students assumed that teachers were available to assist with questions or concerns at any time, leading to an unrealistic expectation of 24/7 availability.

On the other hand, all teachers talked about how the system also reduced workload in some cases, for example, because communication between teachers is facilitated. Other positive points mentioned were that teachers can easily maintain contact with parents and students, and that many system features were automated. A teacher elaborated:

It's easy to keep an overview of whether students are present or not, whether they brought their books, and whether they've done their homework. It's also definitely useful to see… if a student is a bit concerning in my class, whether that's also the case in other subjects, and how they're developing in that regard.

### 3.3 Impact on Teachers' Expectations

Teachers talked about how reading up on students' reports (i.e., observations of other teachers about the development, progress, and motivation of a student) from previous years can lead to biased expectations. As a result, teachers indicated that they did not approach new students with a ‘blank slate’ but rather with some initial expectations about their behavior and performance. Parents and teachers also indicated that they expected students to be less forgetful, given that everything is easily accessible at any time in the online system. “I think it's an advantage for teachers that you put the homework on [name monitoring system], so a student can't get away with saying they didn't know. Because you just put it in the system.”

Also, new expectations of teachers towards parents were imposed by using SMS. Teachers expressed their expectation that parents would use SMS to track their child's progress. When issues arise, teachers said they would rely on parents to initiate communication, placing a shared responsibility for addressing academic concerns onto the parents.

### 3.4 Impact on Students' Competence and Performance

According to many teachers, parents, and students, online SMS have short‐ and long‐term impacts on students' competence and performance. In the short term, a system provides an overview to teachers, parents, and students of the students' progress, time schedule, and the homework that needs to be done. This increased transparency helps students stay organized and focused. Teachers and parents can easily monitor progress, identify areas where students need additional support, and intervene promptly. A student discussed the advantages of having their parents involved in their schoolwork: “My parents can help me make a good choice and give advice. After all, it's my first year, and they know some of the tricks.”

However, the long‐term impact of implementing SMS can be less favorable, parents and teachers said, as students may become overly dependent on it. For example, a teacher raised the concern that as teachers enter all homework into SMS, students no longer feel the need to maintain their agendas and therefore may not learn the competence of managing their schedules and responsibilities. Furthermore, some parents indicated that they stepped in to help with the scheduling of the students (e.g., reminding them about homework or exams), which may further lead to students losing their sense of responsibility and competence to manage their agenda. A teacher said:

I think it does hinder things a bit because it doesn't really foster a sense of personal responsibility. Students become very dependent on this system—they no longer need to keep track of their own agendas. The responsibility now lies with the teacher, whether they enter something into the system or not, rather than with the student. In the past, with an agenda, students were responsible for keeping track of everything themselves. That's no longer the case.

### 3.5 Impact on Students' Wellbeing

The interviewed students mainly named positive consequences of SMS with parent portals on their wellbeing. For instance, they spoke about their positive feelings when receiving positive feedback, and how such a system helps to get more help and support from parents. When thinking about the impact of SMS on other students, the interviewed students named some negative effects. Parents and teachers interviewed also identified these two main negative effects: being an excessive focus on school and performance because the systems are 24/7 accessible, and students' fear of parental reactions in response to low grades. One student said, “Some parents really make a big deal out of it when their children get a bad grade.”

The timing of grade publication was often discussed by teachers, parents, and students. Teachers said grades are often posted late, sometimes at 10 p.m. or on weekends, which may cause stress for students. Some teachers chose to upload grades during school hours to minimize stress for students, with some schools having informal agreements to post grades during specific times, such as avoiding exam weeks. A teacher shared:

A lot of students have their phones next to their beds, so at the moment I publish grades, then *PING*, they see their grade, and of course, they start messaging classmates, and that's not what I want. I try to have everything in by 10 PM, and if it gets later—because it sometimes does—I won't publish them right away. This is also to prevent too much distraction and not getting enough sleep afterward.

In contrast, some teachers deliberately chose not to upload grades during school hours. When grades are posted during school time, students receive their notifications during other classes, which can disrupt those lessons as students become eager to check and compare their grades. Teachers shared their annoyances about other teachers' publication of grades during their class hours. However, another teacher was clear about students needing to hear about their grades at school:

Grades belong to the school environment, so it should be clear when the child is at school, not when they are at home. I find it a bit unfortunate because they can't do much with it at home. They probably don't know what grades their classmates received or what the teacher has to say about it. So, they can't really put it into perspective.

Several interviewees discussed the advantages of students receiving their grades at home, emphasizing the comfort and familiarity that this setting provides. On this, one teacher said:

I know that some of the students really like it to be in a familiar environment. If their grade is poor, they would prefer to find that out at home rather than hearing or seeing it at school, where their classmates are present.

### 3.6 Impact on Students' Autonomy

Both parents and teachers expressed concerns about the loss of student autonomy due to parents' access to grades. While some parents and teachers saw it as beneficial—students can't “lie” about grades—others noted that it limits students' ability to take ownership of their academic journey. For example, by choosing when and how to share their grades, learning from their mistakes and developing their own responsibility. A teacher said:

Student themselves should take responsibility. Sometimes you hear that parents help their children a lot with homework—for example, by checking the questions for them or looking up what homework they have. I don't think that's necessarily a good thing; a student should be able to manage it independently as well.

All interviewed parents used the SMS to review their students' grades, despite the possible impact on their autonomy. One parent summarized this concern:

When you zoom out, you should question whether it should be possible for parents to always monitor and micromanage [their children]. My parents certainly didn't do this with me, and I didn't turn out worse because of it—quite the opposite. It's also a reflection of the current time. It's easy to track everything and stay informed. So, the negative impact would be more the helicopter parenting—wanting to know everything, trying to influence everything from the background, and taking on too much responsibility, which prevents children from focusing on their responsibilities.

The interviewed students themselves reported that while they felt freedom overall, certain actions—such as skipping school—were immediately visible in the monitoring system, which they felt could be a disadvantage. One student shared: “You experience freedom, but if you want to skip school, it shows up right away in the system. That might be a small disadvantage.”

### 3.7 Impact on the Parent–Child Relationship

Teachers, parents, and students discussed how constant parental access to grades impacts the parent–child relationship. One student noted that their parents can see all school information, including grades, before the student does. For the interviewed students, the system didn't affect their relationships, as they typically share their good grades proactively.

Parents appreciated the ability to offer more emotional and practical support, staying informed about school activities. However, the system can create tension for students with lower grades or behavioral issues. One teacher mentioned that poor grades often lead to confrontations at home. As a result, teachers are sometimes asked to delay posting grades online to help students avoid punishment. One teacher said: “Some students say things like, ‘Miss, could you please enter the grade after the weekend? That way, I can still go to football practice.’”

## 4 Discussion

This exploratory study examined the views and experiences of different stakeholders—secondary school students, parents, and teachers—with using online SMS with parent portals in the Netherlands. Practically all Dutch secondary schools currently use these systems to monitor their students' behavior and progress 24/7 in real‐time. The findings indicate that the experiences of these stakeholders are diverse and nuanced, reflecting both the potential advantages and disadvantages of the system. For students, the system could affect their autonomy, sense of responsibility, short‐ and long‐term school performance, and overall wellbeing. All parents experienced a positive impact on their relationship with their child, as the system fostered better communication and understanding. For teachers, the system influenced their workload, expectations of students and parents, and sense of responsibility. These findings are elaborated upon in the following paragraphs and connected to the existing body of literature.

A key benefit of the system for teachers is its ability to streamline administrative tasks, addressing the growing workload challenge [17]. Previous studies show that technological solutions such as Robotic Process Automation help reduce teacher burdens, allowing more focus on teaching [18]. Interviewed teachers valued the system's efficient communication with students, parents, and colleagues, as well as its ability to track student progress.

However, teachers also pointed out drawbacks, particularly the increased expectations from students, parents, and colleagues to keep information constantly updated. This may result in additional administrative work, particularly when preparing reports that are publicly accessible, thereby limiting teachers' professional autonomy—teachers' ability to independently make decisions about their teaching methods, priorities, and classroom practices [19]. Increased standardization and external control, often byproducts of systems such as open SMS, can erode professional autonomy, leading to feelings of reduced freedom and agency in their roles [19]. In light of these considerations, the successful implementation of open SMS hinges on finding a delicate balance between leveraging technology to reduce administrative burdens and ensuring it supports, rather than undermines, the professional autonomy and wellbeing of teachers.

The students interviewed had generally positive experiences with the system. They found it helpful for keeping track of assignments and deadlines. However, stakeholders also identified two main drawbacks for students: a loss of autonomy and privacy, and the impact of the (timing of the) publication of grades on student wellbeing. First of all, interviewed participants noted that a parent portal could reduce student autonomy and privacy, particularly for those with poor grades. This aligns with previous research of Miller and colleagues showing complaints about parental “snooping” [4]. According to the self‐determination theory (SDT) of Ryan and Deci [9], SMS may unintentionally limit students' autonomy as they rely more on the system for tracking assignments and grades. While the system can improve communication between parents and students, it may hinder independence and reduce students' competence in managing their responsibilities [20, 21].

Secondly, concerns about the timing of grade publication were discussed. First of all, SMS, by continuously displaying grades and notifications, can amplify performance pressure and induce smartphone addiction [8, 22]. This pressure and increased screen time may result in sleep disturbances, particularly when grades are released close to bedtime, as this can trigger pre‐sleep worrying [8]. Secondly, according to the United Nations Convention on the Rights of the Child (UNCRC; articles 6 and 31) [23], the constant flow of data not only affects sleep but also infringes on students' rights to play and leisure (Article 31 of the UNCRC). Parental access to these systems further complicates matters, potentially undermining the right to development (Article 6 of the UNCRC) by restricting the growth of autonomy and negatively impacting school motivation [20].

### 4.1 Implications for School Health Policy, Practice, and Equity

Both the benefits and drawbacks of SMS should be considered in school health policies and practice. The system's primary function of tracking student progress is beneficial, while the open access for parents seems to cause disadvantages. This study could give rise to conversations about parental access to SMS and how and when this should be given. One example solution, proposed by the researchers, is that parental access to SMS would be limited to specific times, such as four times a year, to maintain student autonomy while allowing parents to support their child. Schools could then create policies around parental access, and system developers could implement limited access as a default setting. Additionally, schools should establish guidelines for the timing of grade publication‐ for example discussing grades in class before posting them online. Allowing teachers to delay grade postings by a few days could support this process. This approach would ensure SMS remains a helpful tool while protecting student autonomy and wellbeing. These recommendations should be integrated within larger existing school health models, such as the Whole School, Whole Community, Whole Child (WSCC) model [24]. This model emphasizes a holistic view of students, schools, and communities. Limiting parental access to SMS, supporting student autonomy, and fostering positive parent–child–school partnerships aligns with the goals of WSCC.

Importantly, the impact of SMS might not be uniform across all students and families. The results suggest that the impact of such systems on students' sense of autonomy, competence, and the parent–child relationship is dependent on the existing family context. In families where supportive and positive parenting practices are already in place—characterized by avoiding helicopter parenting and micromanagement—children and parents can optimally use SMS [25]. Conversely, in families where the use of the system is not grounded in mutual trust and agreement, it can result in students feeling overly monitored and parents may become too controlling [25]. This so‐called overparenting involves excessive and developmentally inappropriate levels of parental involvement and control in a child's life. This can manifest as high levels of protection, monitoring, and information‐seeking, invading the child's privacy, or removing obstacles and solving problems for the child [26]. Previous research has shown that parents who engage in overparenting can hinder the development of students' planning, motivation, and learning skills through a lower level of self‐regulation [27]. As hypothesized by parents and teachers in this study, the presence of SMS with parent portals may encourage overparenting behaviors among parents and foster an overreliance on the system by students. Moreover, in general, digital technology in education can promote equality by making it easier for everyone to participate and communicate, for example between students, teachers, and parents [28]. However, concerns about a digital divide persist, as access to devices, digital materials, and the internet varies between schools and families, even in countries such as the Netherlands where most students have internet access. Differences in how students use devices in their free time—such as whether parents provide educational apps and guidance—can further widen disparities in digital skills and opportunities.

### 4.2 Limitations and Future Directions

This study has several limitations. First, the sample size of interviewed students was relatively small, which raises concerns about data saturation and the representativeness of the findings. Additionally, the students and parents interviewed may not fully reflect the broader student population, as recruitment was based on convenience sampling. This reliance on convenience sampling may have led to self‐selection, with a greater likelihood of involvement from higher‐performing students, more engaged parents, and teachers with sufficient time availability when approached by the university students conducting the interviews. For example, the interviewed students typically mentioned that they received good grades, and the interviewed parents also highlighted that their children generally performed well academically. The discussed possibility of overparenting, for instance, was mainly discussed hypothetically and indirectly by teachers and parents (not students). Moreover, the sample lacked demographic diversity, which limits equity considerations: perspectives of families with different socioeconomic, cultural, and linguistic backgrounds may not have been adequately captured. Future research should address these limitations by including larger, more diverse samples—possibly comparing different ages and academic performance levels—to capture a broader range of experiences. Future work could also include participants from diverse demographic and socioeconomic backgrounds to better understand equity‐related differences in the use and impact of SMS. Quantitative studies are particularly needed to examine the impact of SMS with parent portals on students' wellbeing. Comparative research could focus on schools with varying levels of monitoring strictness or rules to better understand their effects on different subgroups of adolescents. Furthermore, longitudinal studies could provide valuable insights into the long‐term consequences of such systems on academic performance, mental health, autonomy development, and parent–child relationships.

## 5 Conclusions

In conclusion, this exploratory study focusing on the first two years of Dutch secondary education, highlights the context‐specific and multifaceted impact of online SMS with parent portals on students, parents, and teachers. While these systems offer significant advantages in enhancing communication, improving academic tracking, and fostering parental involvement, they also present challenges related to autonomy, privacy, and workload. For teachers, the systems can streamline administrative tasks, but they also raise concerns about increased expectations and diminished professional autonomy. Parents generally appreciate the enhanced communication and involvement in their children's education, although this can lead to privacy concerns and strained relationships if not carefully managed. For students, the systems can be helpful in tracking progress and getting help with homework, and planning. However, the systems were also seen as diminishing students' autonomy and privacy while also contributing to performance pressure and reduced sleep health due to the timing and accessibility of grade publication.

To maximize the benefits and minimize the drawbacks, it is crucial to strike a balance in system design, such as limiting parental 24/7 access and developing a policy about the timing of the publication of grades. By addressing these concerns, SMS can remain powerful tools for supporting student development while safeguarding the autonomy and wellbeing of all stakeholders involved.

## Funding

This work was supported by the ZonMw (05550032110027, 555002022).

## Ethics Statement

The study was approved by the Ethics Committee of Vrije Universiteit Amsterdam (VCWE‐2024‐091).

## Conflicts of Interest

The authors declare no conflicts of interest.

## Data Availability

The data that support the findings of this study are available on request from the corresponding author. The data are not publicly available due to privacy or ethical restrictions.
